# Integrative and quantitive evaluation of the efficacy of his bundle related pacing in comparison with conventional right ventricular pacing: a meta-analysis

**DOI:** 10.1186/s12872-017-0649-4

**Published:** 2017-08-11

**Authors:** Ziqing Yu, Ruizhen Chen, Yangang Su, Xueying Chen, Shengmei Qin, Minghui Li, Fei Han, Junbo Ge

**Affiliations:** 1 Department of Cardiology, Shanghai Institute of Cardiovascular Diseases, Zhongshan Hospital, Fudan University, Shanghai, 200032 People’s Republic of China; 2 Shanghai Medical College, Fudan University, Shanghai, 200032 People’s Republic of China; 3 Key Laboratory of Viral Heart Diseases, Ministry of Public Health, Shanghai Institute of Cardiovascular Diseases, Zhongshan Hospital, Fudan University, Shanghai, 200032 China

**Keywords:** Meta-analysis, His bundle pacing, para-his bundle pacing, Right ventricular pacing, Cardiac function, Left ventricular ejection fraction

## Abstract

**Background:**

Long-term RVP could bring adverse problems to cardiac electro-mechanics and result in inter- and intra-ventricular asynchrony, impaired labor force, and aggravation of cardiac function. HBRP including direct His bundle pacing and para-His bundle pacing was regarded as a novel physiological pacing pattern to avoid devastating cardiac function. This synthetic study was conducted to integratively and quantitatively evaluate the efficacy of His bundle related pacing (HBRP) in comparison with conventional right ventricular pacing (RVP).

**Methods:**

Published studies on comparison of left ventricular ejection fraction (LVEF), left ventricular end diastolic volume (LVEDV), left ventricular end systolic volume (LVESV), New York Heart Association (NYHA) class, inter-ventricular asynchrony, and QRS duration, etc. between HBRP and RVP were collected and for meta-analysis.

**Results:**

HBRP showed higher LVEF (WMD = 3.9%, 95% CI: 1.6% – 6.1%), lower NYHA class (WMD = −0.5, 95% CI: -0.7 – -0.3), WMD of LVESV = −0.1 ml, 95% CI: -3.0 – 2.8 ml), less inter-ventricular asynchrony (WMD = −13.2 ms, 95% CI: -16.4 – -10.0 ms), and shorter QRS duration for long-term (WMD = −36.9 ms, 95% CI: -40.0 – -33.8 ms), however, no significant difference of ventricular volume (WMDLVEDV = −2.4 ml, 95% CI: -5.0 – 0.2 ml; WMDLVESV = −0.1 ml, 95% CI: -3.0 – 2.8 ml) compared to RVP.

**Conclusions:**

The efficacy of HBRP was firstly verified by meta-analysis to date. Compared with RVP, HBRP markedly preserve LVEF, NYHA class, and QRS duration. However, it seemed to have less effect on ventricular volume.

**Electronic supplementary material:**

The online version of this article (doi:10.1186/s12872-017-0649-4) contains supplementary material, which is available to authorized users.

## Background

Conventional right ventricular pacing (RVP) was indicated for bradycardia caused by kinds of etiology. However, amount of studies disclosed long term RVP especially right ventricular apical pacing (RVAP) adversely affected cardiac electro-mechanics and resulted in poor prognosis, for instance, reduction of left ventricular ejection fraction (LVEF), enlargement of cardiac chambers, pulmonary artery pressure increasing, prolongation of QRS duration, impaired exercised capacity, and even high mortality [1–3]. In physiological state, His bundle, left branch bundle (LBB), and right branch bundle (RBB) were activated orderly. Since the conductive speed of LBB was faster than RBB’s, left ventricular myocardium was activated prior to right ventricular myocardium, and this intrinsic order of activation guarantees synchronous motion of ventricular wall and normal cardiac output. However, long-term RVP changed the sequence of dual-ventricle activation and brought anomaly of cardiac function [1]. Even short-term RVP could acutely influence hemodynamics, leading to reduction of maximal and minimum rate of ventricular pressure change with the time (± dp/dt), pulmonary capillary wedge pressure (PCWP), and valvular regurgitation [4–8]. Thus, the phenomenon of RVP deteriorating cardiac function raised concerns to the permanent pacemaker pacing site [9]. It was reported that right ventricular outflow tract (RVOT) pacing and right ventricular septal (RVS) pacing brought less adverse effect compared to RVAP, and the probable mechanism was because of their superiority to right ventricular apex (RVA) anatomically. Thus, pacing at these sites could partially simulate the physiological sequence of activation [10–12]. Moreover, cardiac resynchronization therapy (CRT) as a dual-ventricle pacing mode was thought to be more favorable to reduce intra-ventricular and inter-ventricular asynchrony [13, 14]. Long-term RVP patients with impaired cardiac function were indicated for CRT upgrading [15, 16]. Nevertheless, the rate of CRT implantation stayed in an unsatisfactory level for its relatively heavy cost and non-response proportion [13]. Anatomically, His bundle lying between atrioventricular node and branch bundles played an important role in cardiac electric conduction [17, 18]. It suggested that stimulation right at His bundle should most closely simulate physiological conduction avoiding myocardial activation in advance. However, in clinical practice, direct His bundle pacing (DHBP) couldn’t be always attained, and para-His bundle pacing (PHBP), a partial His bundle capture, was comparable to DHBP [19, 20]. In present study, DHBP and PHBP were regarded as His bundle related pacing (HBRP). As relatively large and prospective clinical studies of HBRP came out, the so-called physiological pacing draws lots of attention and interest. However, it still lacked study to systemically summarize existing clinical trials and comprehensively evaluate the effects of HBRP. Consequently, present study aimed to do a meta-analysis to collect and analyze published clinical studies of HBRP integratively and quantitatively.

## Methods

### Search strategy and selection criteria

Design and implement of this meta-analysis was abided by a guideline which was broadly adopted [21]. Databases including Pubmed, Web of Science, Ovid, EBSCO, and Cochrane Library were applied with key words (His bundle pacing, Hisian pacing, para-His bundle pacing, and para-Hisian pacing) to retrieve related literatures before October 17th 2016. Clear criteria of successful direct His-bundle pacing or para-His bundle pacing were thought necessary for related literatures. Besides, based on different parameters we aimed to analyze, these already included studies were further divided into subgroups for further analyses. These parameters included left ventricular ejection fraction (LVEF), left ventricular end diastolic volume (LVEDV), left ventricular end systolic volume (LVESV), New York Heart Association (NYHA) class, 6-min walk test (6MWT), mitral regurgitation index (MRI), pulmonary artery systolic pressure (PASP), intra-ventricular asynchrony, pacing threshold (Pth), lead impedence (LI), and myocardial performance index (MPI), also known as Tei index. Literatures were filtrated by criteria as follows. Inclusion criteria: (1) the object was human; (2) clear definition of successful HBRP and direct comparison between HBRP and RVP (The definition of His bundle related pacing included DHBP and PHBP. DHBP: was characterized by the same morphology of QRS-wave and T-wave as the one of sinus rhythm; interval between pacing signal and the initiation of QRS-wave was virtually identical to the His-ventricular interval; lower pacing output only captured his bundle with narrow QRS-wave, but higher pacing output also activated myocardium appearing as pre-excitation like QRS-wave. PHBP: was defined when His potential sensed by the pacing lead was identical to the one by the electrophysiological catheter; besides, higher pacing output evidently shortened QRS-wave.); (3) with effective parameters (LVEF, NYHA, Pth, and MPI, etc.) to evaluate differences between pacing patterns. Exclusion criteria: (1) study whose purpose was centered on acutely response to HBRP and with follow-up duration less than 1 months; (2) without RVP as control group; (3) without access to full text except for abstract only.

### Data extraction and quality assessment

Two investigators who were not informed with the protocol of present meta-analysis independently checked the quality and eligibility and collected related data of studies. Quality assessment of prospective cross-over study was based on Cochrane handbook, and eligibility of cross-over study was described as follows: (1) whether the cross-over design was suitable for the permanent pacing condition; (2) were two different pacing conditions stable or fluctuating; (3) was there existing a elution time between two stages of trial; (4) did participants drop out after the first treatment, and not receive the second treatment; (5) was it clear that the order of receiving treatments was randomized [22]. Besides, observational trials were assessed by using key study design components presented in the Newcastle-Ottawa Scale.

### Data synthesis and analysis

Mean value and standard deviation (SD) of a certain parameter (LVEF, NYHA, Pth, and MPI, etc.) were extracted from included clinical studies, and continuous variables of different studies were integrated to calculate weighted mean differences (WMD) or standard mean differences (SMD). The value was collected and calculated as WMD when its corresponding parameter was measured by the same method. When the corresponding parameter was measured by different method, it was calculated as SMD. Pooled-analyses were implemented using fixed-effect models, whereas random-effect models were applied in case of significant heterogeneity across studies. Statistical heterogeneity was measured using the Inverse Variance (I-V) statistics. Sensitivity analyses (exclusion of 1 study at a time) were performed to determine the stability of the overall treatment effects. Additionally, publication bias was assessed using the Begg’s adjusted rank correlation test and shown as funnel plot. All *p* values were 2-tailed, and the statistical significance was set at 0.05. Statistical analyses were performed using Stata software 12.0 (Stata Corp, College Station, Texas).

## Results

### Literature search

**Fig. 1:**
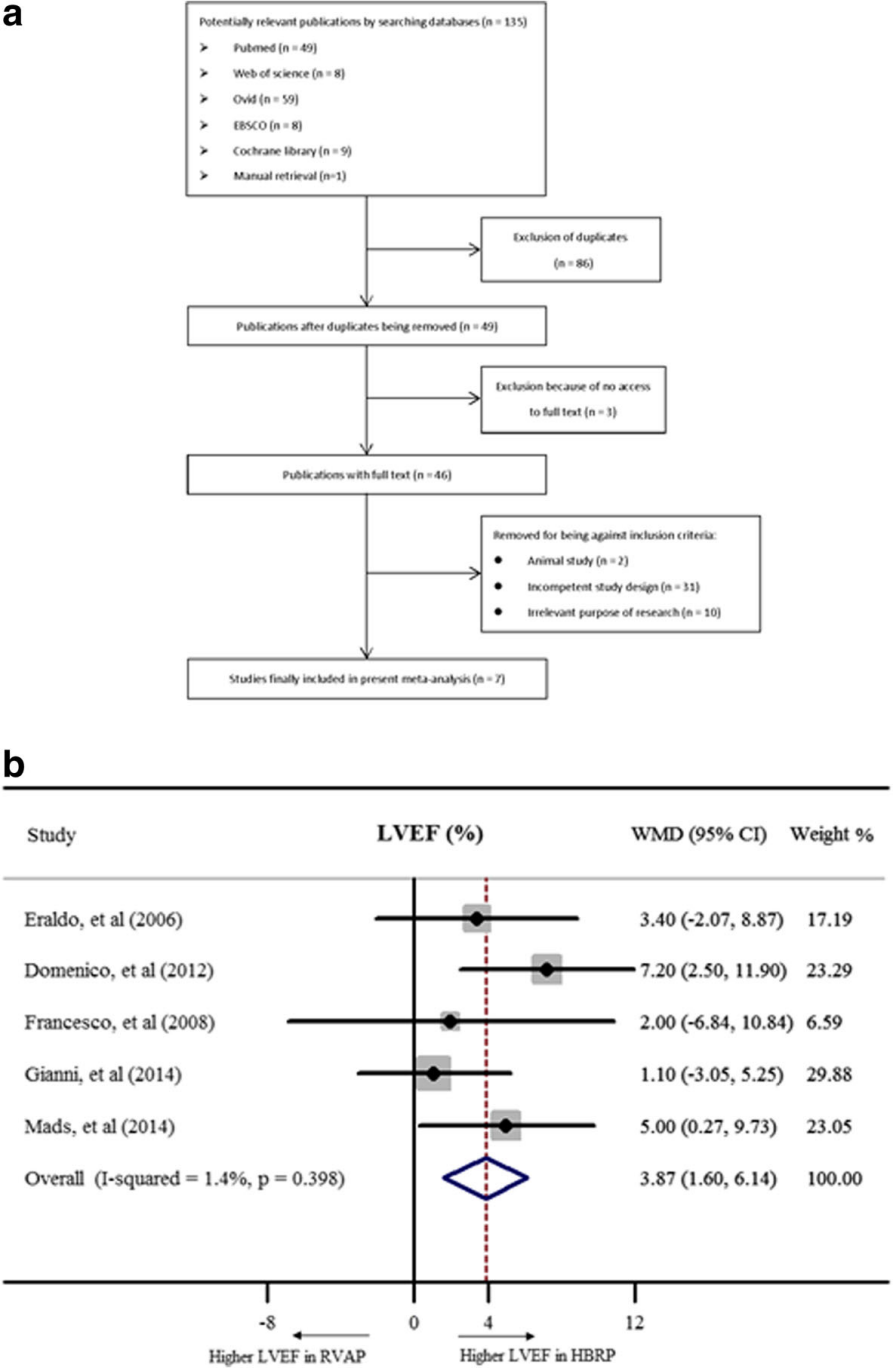
showed flow diagram of the process of clinical studies inclusion and exclusion and left ventricular ejection fraction of HBRP compared to ones of RVP. **a** flow diagram; **b** forrest plot of LVEF; (WMD = weight mean difference, and CI = confidence interval)

Using key words as mentioned before to search different electronic databases, 135 potentially relevant abstracts were yielded. After comparison among different databases, 86 duplicates were removed. Furthermore, 43 literatures were excluded since they didn’t fit the inclusion criteria. 3 of them were eliminated because full text couldn’t be attained. Besides, 2 articles were excluded as animal experiments. 10 case-report studies and 2 case series studies were also removed. 8 studies aimed to analyze the acute response of hemodynamics to HBRP were then excluded. 5 studies concentrated on the comparison of HBRP with CRT. 6 studies were designed as self-contrast method. Instead of HBRP, other 10 studies were related to electrophysiology examination in arrhythmia study. When screening full text of each literature, manual search by checking the reference list helped to further identify one literature. Consequently, 7 studies [20, 23–28] were finally included for meta-analysis finally (Fig. 1A).

### Characteristics and quality assessment of eligible studies

A total number of 325 patients (62% male) were included in the final analysis, and mean value of followed-up duration was over 13 ± 11 months with median of 12 months. In general, the morbidity of concurrent heart disease with ischemic etiology, hypertension, and diabetes were 20%, 46%, and 38% respectively. Additionally, estimated mean age of patients in pooled studies was 71.5 ± 8.4 years old. Basic elements of clinical studies, such as main investigator, year of publication, regions, study design, duration of follow-up, and number of patients included in present meta-analysis were shown in Table 1. Since none of parameters which were used to evaluate the difference between HBRP and conventional RVP could be collected from whole the pooled studies, characteristics of each subgroup referred to the relevant parameter and the significance of difference were exhibited in Table 2. Quality assessment of included literatures was shown in Table 3.

**Table 1:** General description of included clinical studies

Author	Year	Region	N of patients	Selection of patients	Study design	Median follow-up duration	DHBP of total HBRP (%)	N of HBRP implantation failure
Eraldo, et al.	2006	Italy	197	AF	Prospective crossover blinded randomized controlled study	6 months	0 (0)	1
Kenneth, et al.	2015	America	173	unselected	Observational study	24 months	34 (45)	NG
Domenico, et al.	2012	Italy	26	nonHF	Prospective crossover cohort	34 months	20 (76.9)	NG
Francesco, et al.	2008	Italy	12	nonHF	Prospective crossover cohort	6 months	12 (100)	NG
Gianni, et al.	2014	Italy	37	nonHF	Prospective crossover cohort	3 months	17 (46)	0
Mads, et al.	2014	Denmark	38	nonHF	Prospective crossover blinded randomized controlled study	12 months	4 (10.5)	3
Domenico, et al.	2006	Italy	24	unselected	Prospective crossover cohort	7 months	17 (73.9%)	1

*AF* atrial fibrillation, *N* number, *DHBP* direct his bundle pacing, *HBRP* his bundle related pacing, *NG* not given

**Table 2:** Demographic characteristics of patients at baseline

Author (year)	Age (year)	Male (%)	NYHA class	QRS (ms)	QRS < 120 ms (%)	LVEF (%)	LVEF < 50% (%)	IHD (%)	AF (%)	Hypertension (%)	DM (%)	β-blocker (%)	CCB (%)	Diuretics (%)	ASA (%)	ACEI/ARB (%)	Digoxin (%)
Eraldo, et al. (2006)	71.4 ± 5.6	50	2.33 ± 0.6	88.3 ± 7.1	100	52 ± 9.1	5	4	16	6	NG	31	19	NG	12	75	56
Kenneth, et al. (2015)	74 ± 12	58	NG	109 ± 26	NG	56 ± 9	0	NG	NG	NG	NG	NG	NG	NG	NG	NG	NG
Domenico, et al. (2012)	71.6 ± 8.8	61.5	NG	97.7 ± 11.8	NG	57.2 ± 7.4	0	37	NG	38	NG	69	39	73	46	50	8
Francesco, et al. (2008)	74 ± 9	75	1.6 ± 0.7	86.2 ± 18.8	100	59.8 ± 7	0	NG	NG	NG	NG	NG	NG	NG	NG	NG	NG
Gianni, et al. (2014)	67.4 ± 7.3	70	NG	157.8 ± 14.2	100	66.3 ± 8	0	16.2	NG	43.2	8.1	NG	NG	NG	NG	NG	NG
Mads, et al. (2014)	67 ± 10	79	1.4 ± 0.7	93 ± 16	100	55 ± 10	0	11	3	50	21	18	NG	13	NG	26	NG
Domenico, et al. (2006)	75.1 ± 6.4	61.5	NG	97.7 ± 11.8	100	57.2 ± 7.4	0	37	NG	38	NG	69	39	73	46	50	8

*NYHA* New York Heart Association, *LVEF* left ventricular ejection fraction, *IHD* ischemic heart disease, *AF* atrial fibrillation; DM: diabetes mellitus, *CCB* calcium channel blocker, *ASA* aspirin, *ACEI/ARB* Angiotensin-Converting Enzyme Inhibitors / Angiotensin Receptor Blocker, *NG* not given

**Table 3:** Quality assement of eligible literatures

Quality Assesment								
Cross-over study	Author	Prospective design	Clear definition of study population	(1)	(2)	(3)	(4)	(5)
	Eraldo, et al.	Yes	Yes	Yes	Stable	No	No	Randomized
	Domenico, et al.	Yes	Yes	Yes	Stable	No	No	Not clear
	Francesco, et al.	Yes	Yes	Yes	Stable	No	No	Not clear
	Gianni, et al.	Yes	Yes	Yes	Stable	No	No	Not clear
	Mads, et al.	Yes	Yes	Yes	Stable	No	No	Randomized
	Domenico, et al.	Yes	Yes	Yes	Stable	No	No	Not clear
Observational study	Author	Study design	Clear definition of study population	Clear definition of different pacing modes	Clear definition of related endpoints	Blindness to a certain pacing mode	Representativeness of the study population	Comparability between case and control groups
	Kenneth, et al.	Observational	Yes	Yes	Yes	Not clear	Yes	Yes

(1) whether the cross-over design was suitable for the permanent pacing condition; (2) were two different pacing conditions stable or fluctuating; (3) was there existing an elution time between two stages of trial; (4) did participants drop out after the first treatment, and not receive the second treatment; (5) is it clear that the order of receiving treatments was randomized

### Preservation and improvement of cardiac function with HBRP

**Fig. 2:**
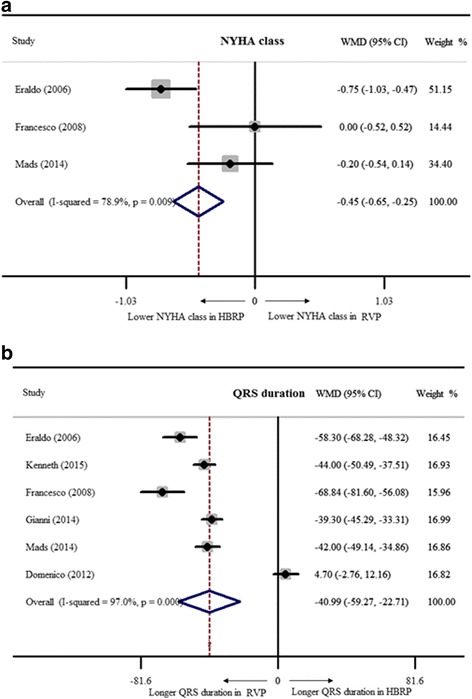
New York Heart Association class and QRS duration of HBRP compared to ones of RVP. **a**. NYHA class; **b**.QRS duration

**Fig. 3:**
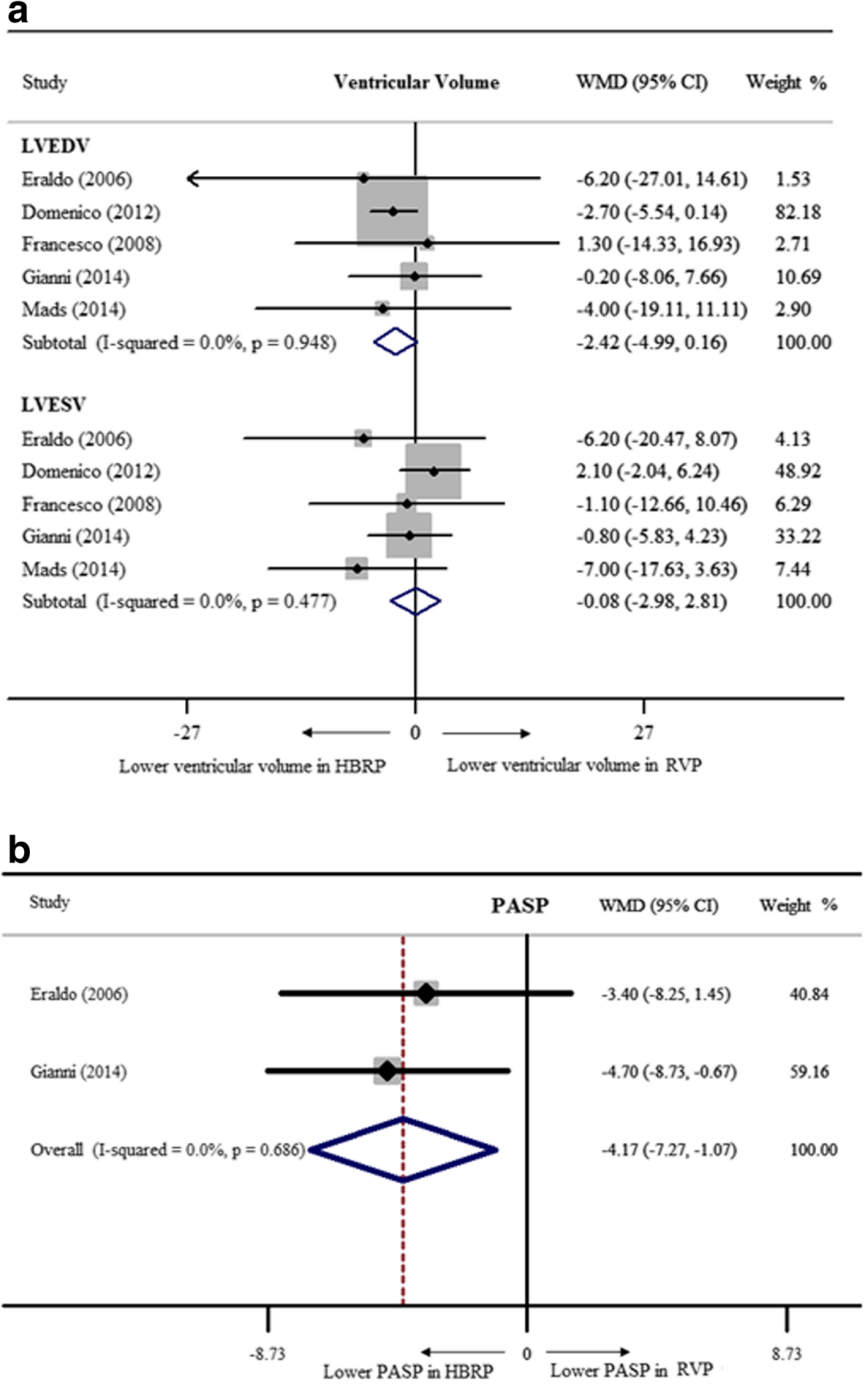
Ventricular volume and pulmonary artery systolic pressure of HBRP compared to RVP’s. **a** showed lower ventricular volume in HBRP; **b** showed lower PASP in HBRP

**Fig. 4:**
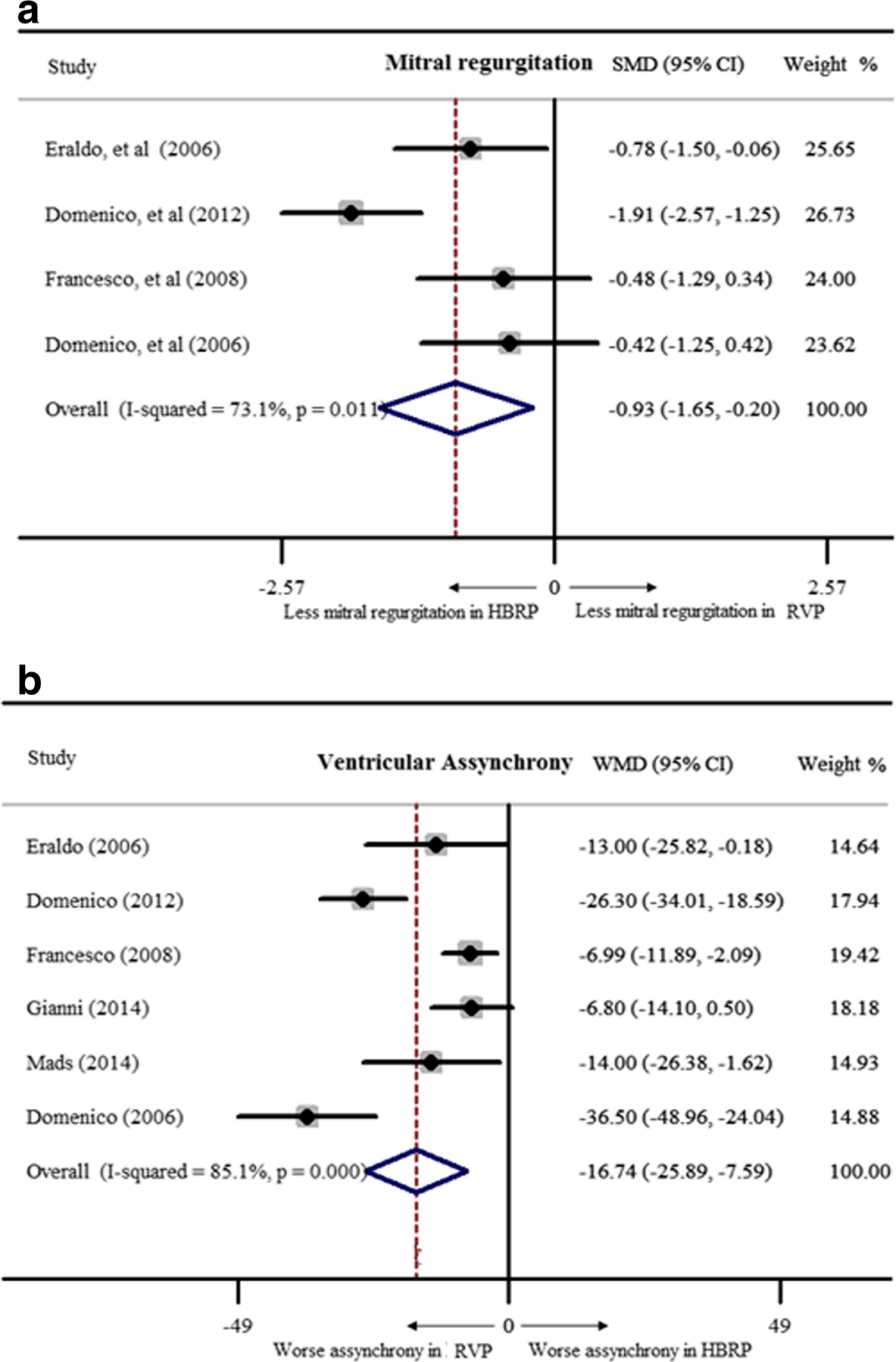
Mitral regurgitation and ventricular assynchrony of HBRP compared to RVP’s (SMD = standard mean difference). **a** showed less mitral regurgitation in HBRP; **b** showed worse ventricular asynchrony in RVP

**Fig. 5:**
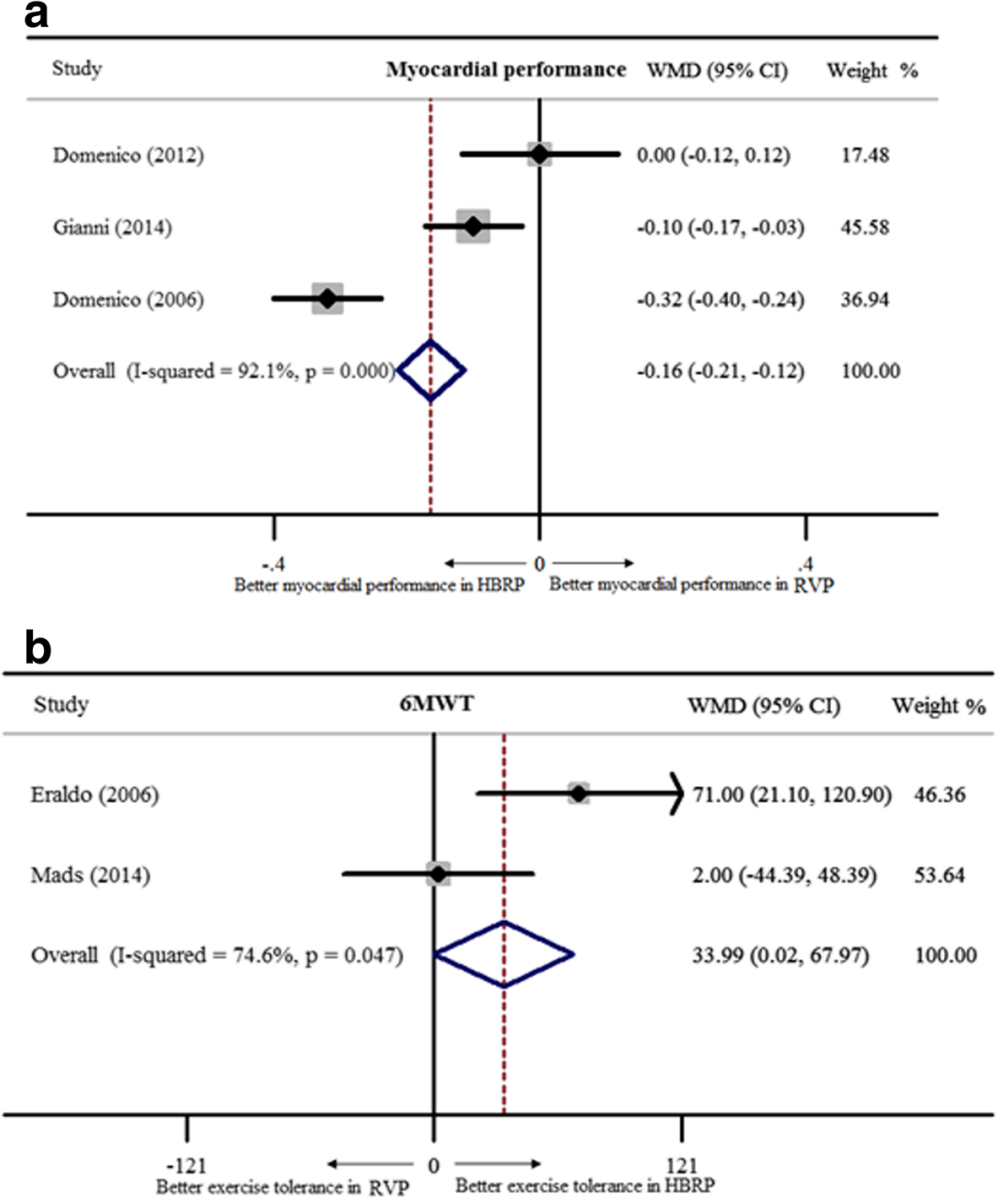
Myocardial performance index and 6-min walk test of HBRP compared to RVP’s. **a** showed better myocardial performance in HBRP; **b** showed better exercise tolerance in HBRP

For the effect of different pacing pattern on LVEF, 5 studies were finally included with 129 patients. Fixed-effect model was applied with *I* ^*2*^ = 1.4% indicating good homogeneity among studies. LVEF was markedly preserved in patients with HBRP (WMD = 3.9%, 95% CI: 1.6% – 6.1%, Fig. 2A). 3 studies with 66 patients suggested a lower NYHA class in HBRP by a fixed-effect model (WMD = −0.5, 95% CI: -0.7 – -0.3, Fig. 2B). For evaluating left ventricular volume, 5 studies with 129 patients were included, and it showed no significance (WMD of LVEDV = −2.4 ml, 95% CI: -5.0 – 0.2 ml; WMD of LVESV = −0.1 ml, 95% CI: -3.0 – 2.8 ml, Fig. 3A). PASP was analyzed with 53 patients from 2 studies by a fixed-effect model, revealing lower PASP in HBRP (WMD = −4.2 mmHg, 95% CI: -7.3 – -1.1 mmHg, Fig. 3B). The way to semi-quantitatively estimate the degree of MR was different among 4 studies with 77 patients, and SMD calculated by a random-effect model indicated that HBRP obviously alleviated MR (SMD = −1.0, 95% CI: -1.4 – -0.6, Fig. 4A). To evaluate the difference of inter-ventricular asynchrony, 6 studies with 152 patients were included, and heterogeneity among studies was proved (*I* ^*2*^ = 85.1%). Thus, a random-effect model was adopted to show better inter-ventricular synchronized motion in HBRP (WMD = −13.2 ms, 95% CI: -16.4 – -10.0 ms, Fig. 4B). Comparison of MPI between 2 pacing modes with 3 studies including 86 patients in a random-effect model indicated better myocardial performance in HBRP (WMD = −0.16, 95% CI: -0.21 – -0.16, Fig. 5A). Besides, there were 2 studies with 54 patients to show better performance of 6MWT in HBRP (WMD = 34 m, 95% CI: 0–68.0 m, Fig. 5B).

### Influence of pacing pattern on QRS complex duration

From 6 studies with 301 patients, QRS duration after long-term HBRP was longer than intrinsic QRS duration in a fixed-effect model (WMD = 4.3 ms, 95% CI: 1.4–7.3 ms, Fig. 2B), and this went against the viewpoint that His bundle or para-His bundle pacing didn’t widen QRS duration [29]. In addition, from 6 studies with 302 patients after different pattern of pacing with equal QRS duration at baseline, RVP showed a distinctly longer QRS duration than the one of HBRP in a random-effect model (WMD = −36.9 ms, 95% CI: -40.0 – -33.8 ms (Additional file 1: Figure S1).

### Influence of pacing pattern on intrinsic parameters of pacemaker in acute stage

Compared to RVP, exposure time to fluoroscopy during procedure of RVP was longer (WMD = 5.7 min, 95% CI: 3.4–8.1 min, Additional file 1: Figure S2A). Moreover, HBRP had lower pacing LI (WMD = −88.2 Ω, 95% CI: -112.4 – −64.0 Ω, Additional file 1: Figure S2B). Additionally, Pth measured in pacemaker implantation procedure showed HBRP needed higher Pth (WMD = 0.92 V*0.5 ms, 95% CI: 0.78–1.06 V*0.5 ms Additional file 1: Figure S3A). What’s more, in comparison with RVP, amplitude of R wave in ECG was lower (WMD = −6.321 mV, 95% CI: -7.659 – -4.984 mV, Additional file 1: Figure S3B).

### Analysis of sensitivity and publication bias

Sensitivity analyses suggested that difference of parameters of cardiac function (e.g. LVEF and cardiac asynchrony, etc.) was concealed when one of included studies was omitted (Additional file 1: Table S1). However, since number of some studies included for analysis of a certain parameter was less than three, sensitivity analysis might not suitable in such a situation. There was no publication bias of different parameter being found in included clinical studies by Begg’s test with all *p* values >0.05 and shown as horizontal funnel plot in Additional file 1: Figure S4.

## Discussion

On account of small scale but considerable importance of existed clinical studies on HBRP, meta-analysis was especially suitable for comprehensively analyzing the outcomes of HBRP. To our knowledge, present study was the first meta-analysis to systemically and quantitatively evaluate the effect of HBRP. Findings of this study were as follows: (1) LVEF was significantly higher in HBRP patients than the one in RVP patients; (2) NYHA class in HBRP groups was lower; (3) in addition, HBRP was superior to RVP in MPI; (4) left ventricular volume (both LVESV and LVEDV), however, showed no significant difference between two pacing patterns; (5) inter-ventricular synchronized motion was preserved by HBRP; (6) mitral regurgitation was alleviated by HBRP; (7) HBRP resulted in lower PASP and better exercise tolerance; (8) QRS duration presented remarkable elongation after months of RVP compared to HBRP, however, long-term HBRP had slightly but significantly widened QRS complex; (9) HBRP was different from RVP on Pth, lead impedence, RWA and FET during procedure. Pth in HBRP increased distinctly from the one in RVP, while impedence of pacing lead in HBRP was markedly lower than the one in RVP. Our findings firstly disclosed the significant predominance of HBRP over RVP by meta-analysis. From this study, HBRP was confirmed to preserve cardiac conduction and corrected cardiac asynchrony. Thus, the performance of heart was improved, and the overall situation of patients was even better. However, LVESV and LVEDV showed no difference between two pacing modes, and this might be related to limited follow-up duration. It was reported that cardiac dysfunction emerged prior to structural abnormality in RVP related cardiomyopathy [30]. Hence we reckoned that prolonged follow-up duration of different pacing sites should attain significant difference of ventricular volume. It was reported that long-term RVP resulted in prolonged QRS complex duration compared with the one at baseline [31]. Nevertheless, QRS complex duration at baseline was not equal to the one after HBRP for a long time (median value of follow-up was 12-month). It hinted that though HBRP was regarded as physiological pacing pattern, it might still widen QRS complex somehow. The reason that Pth value was higher in early stage of HBRP might result from abundant fibrous structure instead of myocardial tissue [19, 32]. Besides, persistent high level of Pth lead to faster battery consumption, and this could incur earlier battery depletion and replacement. HBRP with injury current recorded when mapping His bundle indicated lower Pth than the one in HBRP without injury current [33]. Thus, injury current might indicate better His bundle capture and longer lifetime of pacemaker. Considering lower lead impedence in early phase of HBRP, resistance would outstandingly reduce when pacing electrode was directly placed at His bundle [20, 24, 25]. However, lacking of relevant data, we failed to analyze the change of both Pth and impedence after long-term follow-up.

As conventional RVP brought undesirable problems, heart physicians thought deeply about introducing a brand-new substitute pacing mode. Early researche indicated that patients could benefit more from pacing site at RVOT or RVS than RVP [10, 12], and this was further confirmed by meta-analyses [34–36]. In spite of significant difference compared to RVP, RVOT/RVS pacing was not enough for physiological pacing pattern [17]. Physiological pacing drew a lot of attention, since Deshmukh et al. originally verified the efficacy and safety of HBRP in a 18-patient cohort [28]. It was reported that HBRP could avoid activating ventricular myocytes before impulse really reach the Purkinje fiber, and hence meet the requirement of physiological pacing. Besides, HBRP could outstandingly improve the synchronization of ventricular electro-mechanics [37–39]. What’s more, HBRP showed equal even better effect compared with CRT in ejection fraction reduced heart failure (EFrHF) patients with or without left bundle branch block and widening QRS complex. Particularly in patients with narrow QRS complex and non-left bundle branch block who were not strongly indicated for CRT implantation [19, 38], HBRP showed greater effect. Besides, the ventricular activation sequence in HBRP was different from CRT. CRT characterized by left ventricle depolarizing from epicardium to endocardium and repolarizing from endocardium to epicardium. This went against physiological condition and originated transmural electrophysiological heterogeneities underlying arrhythmogenesis in early phase of CRT implantation [40–42]. Thus, HBRP could preserve cardiac function, as well as preventing from cardiac arrhythmia. Basic research on canine model suggested that pacing site on higher spetum could reduce cardiac electrical remodeling [43], and His bundle pacing could narrow QT interval and restore electrical synchronization [44]. Moreover, HBRP electrode could be placed without passing through tricuspid valve, resulting in obviously reduced incidence of tricuspid regurgitation and injury [17, 23]. RVP could lead to atrial fibrillation (AF), because RVP increased the dispersion and heterogeneity of atrial electro-mechanics [3]. However, included clinical studies mentioned above didn’t report the occurrence of new-onset AF. Therefor comparison of AF occurrence between RABP and HBRP couldn’t be assessed in this meta-analysis.

In spite of advantages of HBRP, there were some problems in the course of procedure. First of all, potential mapping of His bundle was necessary, and it needed further training for heart physician to acquire this technique. In addition, electrophysiological examination might make it longer for the procedure of HBRP implantation, as well as exposing to more radiation [20, 24]. Currently, it was common to place another back-up electrode at right ventricular in case of dislocation of HBRP electrode. However, it was reported that back-up RVP wasn’t indispensable for HBRP [45]. Besides, extra pacing lead meant higher risk of device related complication and more medical expense, therefore clinician should pay attention to these potential problems. Considering the equal efficacy of implantable cardioverter defibrillator (ICD) electrode positioned to either septum or apex [46–48], leads of HBRP and ICD could be then integrated. Furthermore, QRS duration in some patients with LBBB wasn’t shortened by HBRP because of infra-His bundle block close to branch bundles [17, 49], and hence, distal His bundle pacing should be preferred for fear of His bundle block in proximal and medial sites.

### Limitation

This meta-analysis included 7 clinical studies to evaluate the efficacy and safety of HBRP compared to RVP. Though included clinical studies were based on prospective cohorts in the majority, the number of patients was limited, and the methods of randomization and blindness were not clearly given. Besides, with deficiency of other important parameters, such as time of exposure to fluoroscopy, duration of operation, and long-term Pth and impedence, our study failed to integratively assess these indices. Another point that shouldn’t be ignored was that current HBRP clinical studies were most designed as cross-over study, however, the elution time before switch from one pacing mode to another was not yet possible since patients with indication for permanent pacemaker had to receive continuous pacing without interruption. In addition, evaluation of cardiac asynchrony in included studies was mostly by inter-ventricular mechanical delay, however intra-left ventricular asynchrony contributed more to cardiac dysfunction [50]. Consequently, large scale, prospective, multi-center, double-blind, and randomized parallel-controlled trials are still highly needed.

## Conclusion

To date, present study was the first meta-analysis quantitatively verifying the superiority of HBRP over RVP. From this meta-analysis and review of literatures, we demonstrated the efficacy of HBRP. RVP could definitely lead to or aggravate cardiac dysfunction. Oppositely, either direct His bundle pacing or para-His bundle pacing could preserve or improve cardiac function (LVEF, NYHA class, myocardial performance index, and 6MWT), inter- and intra- ventricular synchrony, ventricular volume, and narrow QRS complex. Thus, HBRP should be a promising pacing pattern in future. However, there existed some problems might potentially limit the application of HBRP. First of all, HBRP needed higher Pth, and it might result in short lifetime of battery. Secondly, His bundle potential mapping needed further training of electrophysiological examination for physician. Besides, this procedure could increase time of exposure to fluoroscopy. In addition, considering the possibility of HBRP electrode dislocation, an extra back-up RVP electrode might need, which could increase hospitalization costs and pacemaker related adverse events (such as infection and tricuspid regurgitation, etc.). Nevertheless, HBRP should be firstly suggested unless extra indication for RVP (e.g. hypertrophic obstructive cardiomyopathy whose outflow tract obstruction could be alleviated by RVP) existing. At last but not at least, although present meta-analysis of HBRP threw light on physiological pacing, technological improvement of HBRP and more prospective, large scale, double-blind, randomized, controlled, and multi-center collaborative clinical trials were still in high need in future.

## Additional files

Additional file 1 **Figure S1.** QRS duration of HBRP compared to the one at baseline: this figure showed the difference between post long-term HBRP QRS duration and intrinsic QRS duration at baseline (WMD = weight mean difference, and CI = confidence interval). **Figure S2.** Fluoroscopic time and lead impedence of HBRP compared to RVAP’s: this figure showed fluoroscopic time and lead impedence of HBRP compared to RVAP’s. A showed higher dose of radiation in HBRP during the procedure; B showed lower lead impedence in HBRP measured during the procedure. **Figure S3.** Pacing threshold and pacing R wave amplitude of HBRP compared to ones of RVAP: this figure showed pacing threshold and pacing R wave amplitude of HBRP compared to ones of RVP. A showed pacing threshold of HBRP different from the one of RVP during procedure; B showed difference of amplitude of R wave between 2 different pacing patterns. **Figure S4**: this figure presented horizontal funnel plots for test of publication bias: A. LVEF; B. LVEDV; C. LVESV; D. inter-ventricular asynchrony; E. NYHA class; F. mitral regurgitation; G. myocardial performance index (Tei index); H. 6MWT; I. PASP; J. QRS duration (HBRP versus baseline); K. QRS duration (HBRP versus RVP); L. fluoroscopy exposure time; M. lead impedence; N. pacing threshold; O. amplitude of R wave. **Table S1.** Sensitivity analysis of different group of studies for each parameter: this table showed sensitivity analyses by the way of exclusion of 1 study at a time to determine the stability of the overall treatment effects (LVEF = left ventricular ejection fraction, LVEDV = left ventricular end diastolic volume, LVESV = left ventricular end systolic volume, NYHA = New York Heart Association, MPI = myocardial performance index, 6MWT = 6 min walk test, PASP = pulmonary artery systolic pressure, Pth = pacing threshold, and RWA = R wave amplitude) (DOCX 355 kb)

## Abbreviations

6MWT: 6-min walk test (6MWT)
DHBP: direct His bundle pacing
HBRP: His bundle related pacing
LVEDV: left ventricular end diastolic volume
LVEF: left ventricular ejection fraction
LVESV: left ventricular end systolic volume
NYHA: New York Heart Association
PHBP: para-His bundle pacing
RVA: right ventricular apical
RVOT: right ventricular outflow tract
RVP: right ventricular pacing
