# Supplementary material for: Integrative and quantitive evaluation of the efficacy of his bundle related pacing in comparison with conventional right ventricular pacing: a meta-analysis
Source: BMC Cardiovasc Disord. 2017 Aug 11;17:221. doi: 10.1186/s12872-017-0649-4 (PMC5553603; doi:10.1186/s12872-017-0649-4)
Supplement: Supplementary file 1 — Figure S1. QRS duration of HBRP compared to the one at baseline: this figure showed the difference between post long-term HBRP QRS duration and intrinsic QRS duration at baseline (WMD = weight mean difference, and CI = confidence interval). Figure S2. Fluoroscopic time and lead impedence of HBRP compared to RVAP’s: this figure showed fluoroscopic time and lead impedence of HBRP compared to RVAP’s. A showed higher dose of radiation in HBRP during the procedure; B showed lower lead impedence in HBRP measured during the procedure. Figure S3. Pacing threshold and pacing R wave amplitude of HBRP compared to ones of RVAP: this figure showed pacing threshold and pacing R wave amplitude of HBRP compared to ones of RVP. A showed pacing threshold of HBRP different from the one of RVP during procedure; B showed difference of amplitude of R wave between 2 different pacing patterns. Figure S4: this figure presented horizontal funnel plots for test of publication bias: A. LVEF; B. LVEDV; C. LVESV; D. inter-ventricular asynchrony; E. NYHA class; F. mitral regurgitation; G. myocardial performance index (Tei index); H. 6MWT; I. PASP; J. QRS duration (HBRP versus baseline); K. QRS duration (HBRP versus RVP); L. fluoroscopy exposure time; M. lead impedence; N. pacing threshold; O. amplitude of R wave. Table S1. Sensitivity analysis of different group of studies for each parameter: this table showed sensitivity analyses by the way of exclusion of 1 study at a time to determine the stability of the overall treatment effects (LVEF = left ventricular ejection fraction, LVEDV = left ventricular end diastolic volume, LVESV = left ventricular end systolic volume, NYHA = New York Heart Association, MPI = myocardial performance index, 6MWT = 6 min walk test, PASP = pulmonary artery systolic pressure, Pth = pacing threshold, and RWA = R wave amplitude) (DOCX 355 kb) [file 12872_2017_649_MOESM1_ESM.docx]

Figure S1 QRS duration of HBRP compared to the one at baseline

**
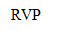

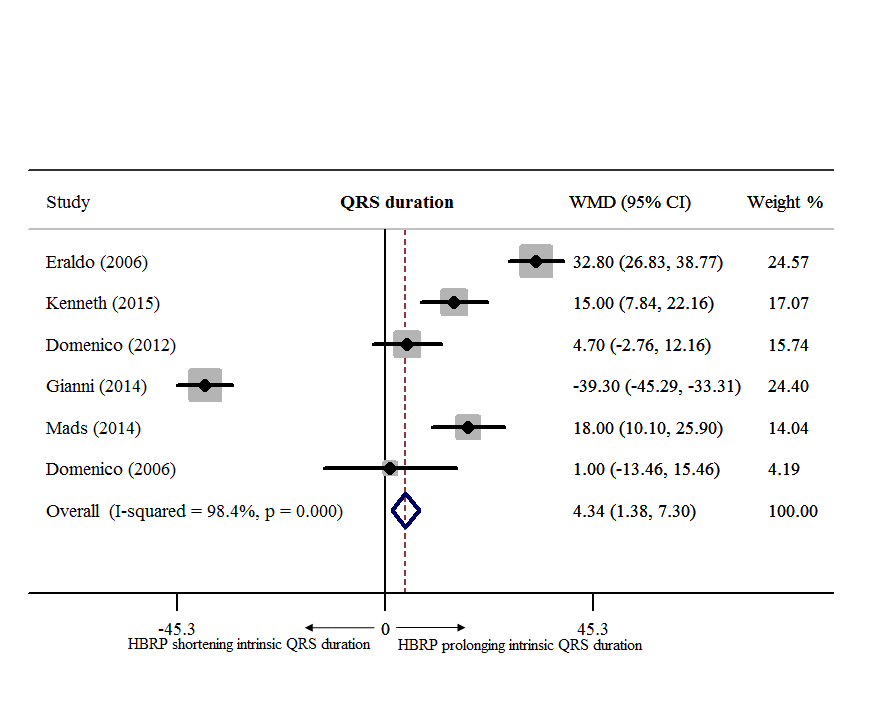
**

This figure showed the difference between post long-term HBRP QRS duration and intrinsic QRS duration at baseline (WMD = weight mean difference, and CI = confidence interval).

Figure S2 Fluoroscopic time and lead impedence of HBRP compared to RVAP’s


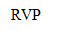
**
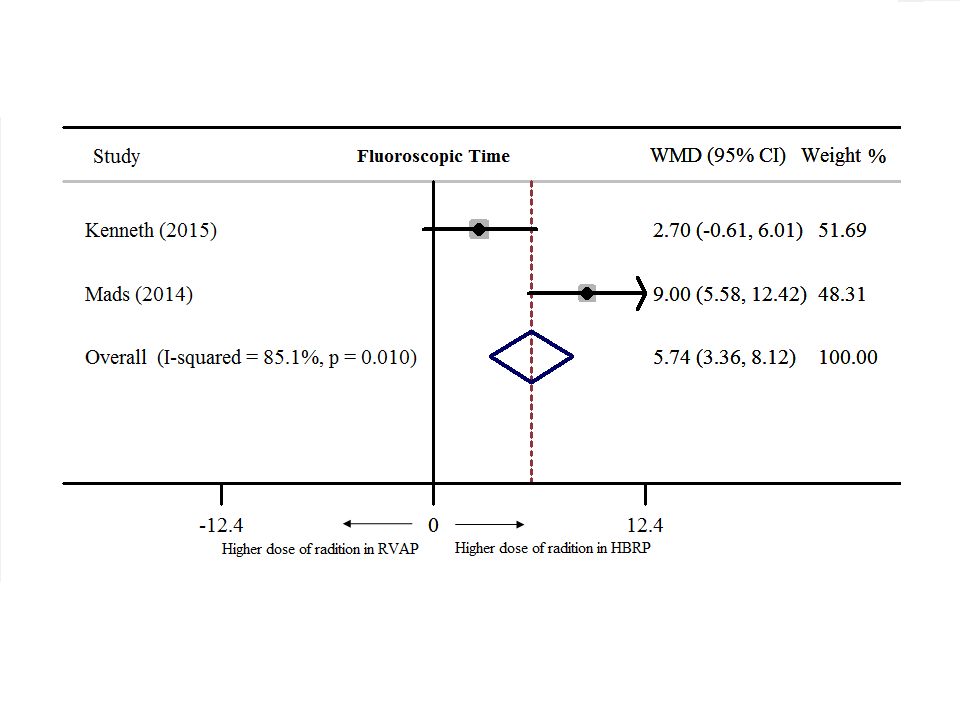
**

**A**


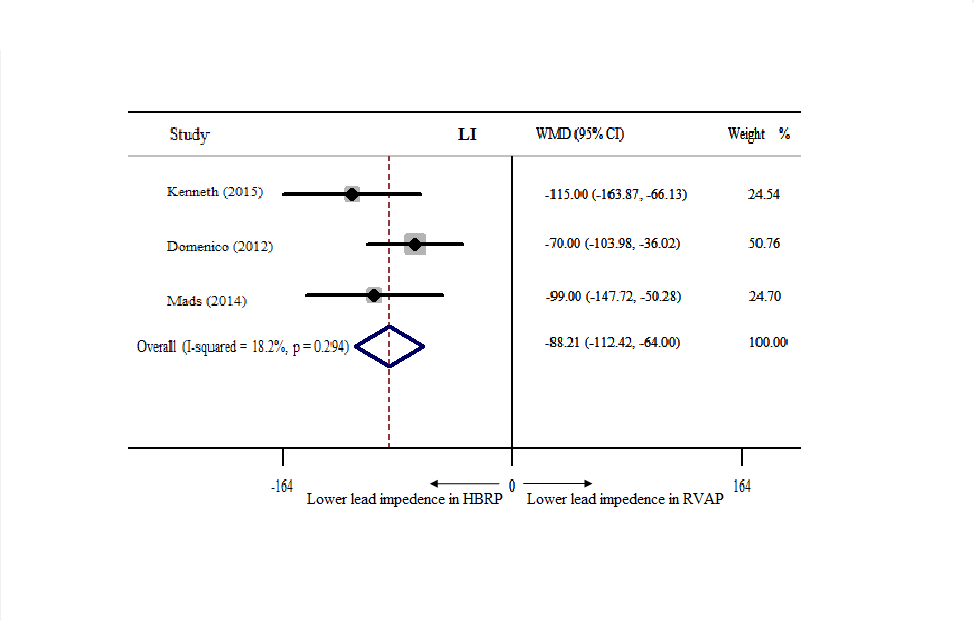


**B**


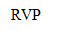


This figure showed fluoroscopic time and lead impedence of HBRP compared to RVAP’s. A showed higher dose of radiation in HBRP during the procedure; B showed lower lead impedence in HBRP measured during the procedure.

Figure S3 Pacing threshold and pacing R wave amplitude of HBRP compared to ones of RVAP


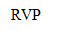
**
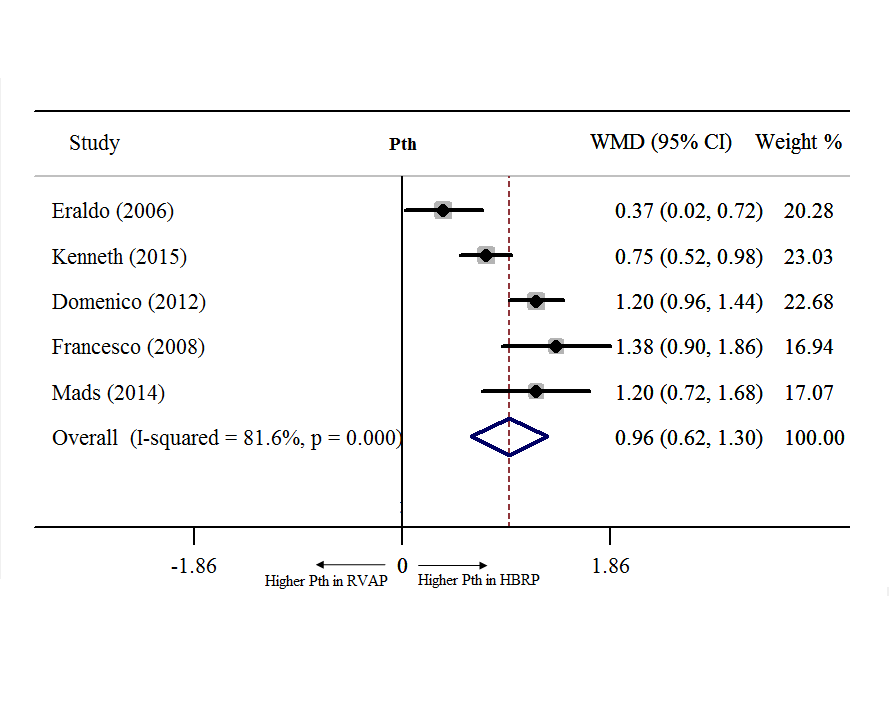
**

**A**


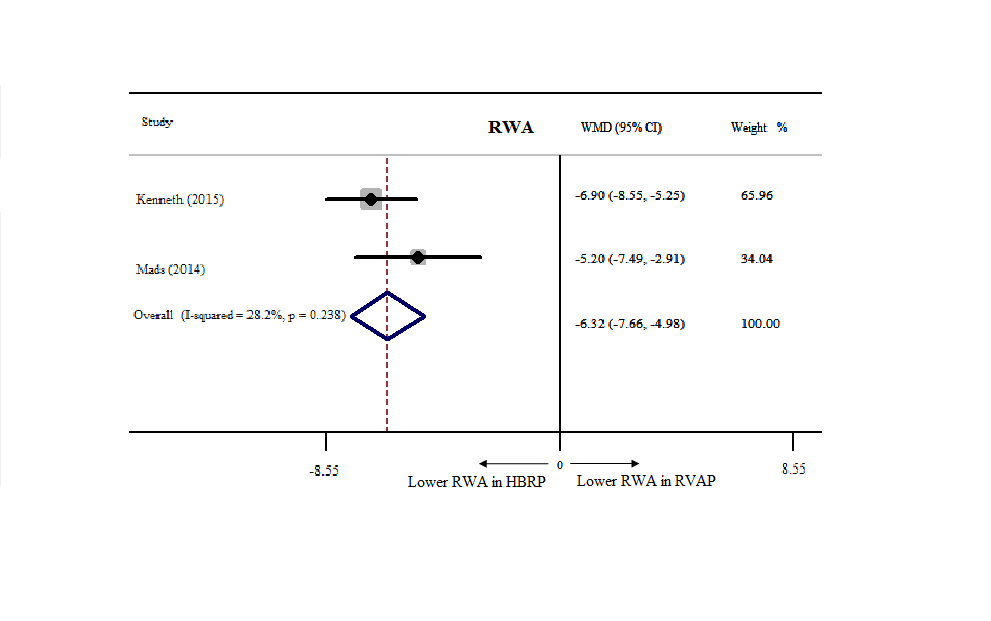


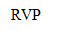


**B**

This figure showed pacing threshold and pacing R wave amplitude of HBRP compared to ones of RVAP. A showed pacing threshold of HBRP different from the one of RVAP during procedure; B showed difference of amplitude of R wave between 2 different pacing patterns.

Figure S4


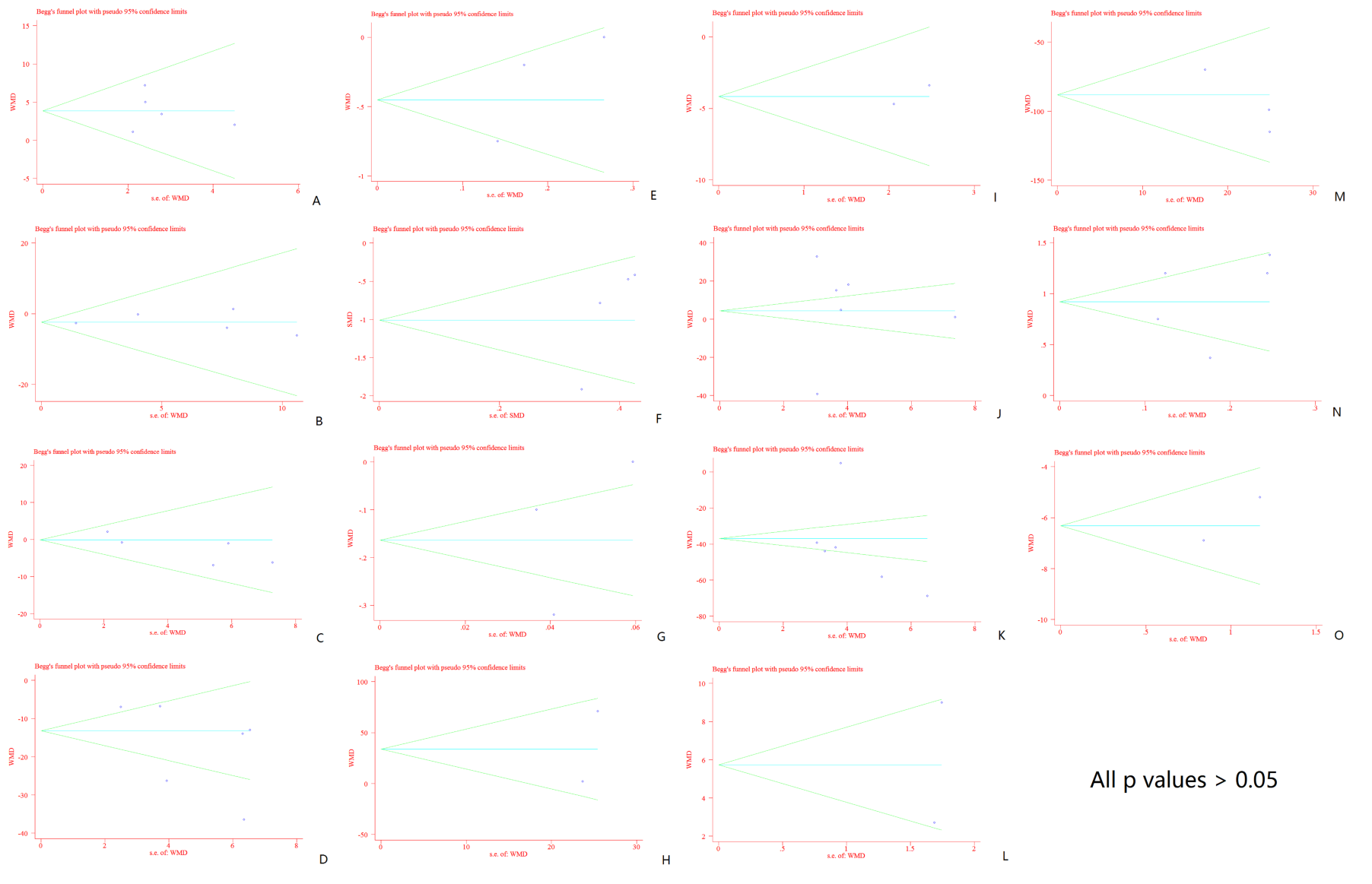


This figure presented horizontal funnel plots for test of publication bias: A. LVEF; B. LVEDV; C. LVESV; D. inter-ventricular asynchrony; E. NYHA class; F. mitral regurgitation; G. myocardial performance index (Tei index); H. 6MWT; I. PASP; J. QRS duration (HBRP versus baseline); K. QRS duration (HBRP versus RVAP); L. fluoroscopy exposure time; M. lead impedence; N. pacing threshold; O. amplitude of R wave.

Table S1. Sensitivity analysis of different group of studies for each parameter

| **Parameters** | **Study omitted** | **Estimate value** | | **95% CI** | | | **Parameters** | **Study omitted** | **Estimate value** | **95% CI** | |
| --- | --- | --- | --- | --- | --- | --- | --- | --- | --- | --- | --- |
|  |  |  |  | **Lower** | | **Upper** |  |  |  | **Lower** | **Upper** |
| **LVEF** | |  | |  | |  | **6MWT** | |  |  |  |
|  | Eraldo, et al (2006) | 3.97 | | 1.48 | | 6.47 |  | Eraldo, et al (2006) | 2.00 | -44.39 | 48.39 |
|  | Domenico, et al (2012) | 2.86 | | 0.27 | | 5.46 |  | Mads, et al (2014) | 71.00 | 21.10 | 120.90 |
|  | Francesco, et al (2008) | 4.01 | | 1.66 | | 6.35 |  | Combined | 33.99 | 0.02 | 67.97 |
|  | Gianni, et al (2014) | 5.06 | | 2.35 | | 7.77 | **PASP** | |  |  |  |
|  | Mads, et al (2014) | 3.54 | | 0.95 | | 6.12 |  | Eraldo, et al (2006) | -4.70 | -8.73 | -0.67 |
|  | Combined | 3.87 | | 1.60 | | 6.14 |  | Gianni, et al (2014) | -3.40 | -8.25 | 1.45 |
| **LVEDV** | |  | |  | |  |  | Combined | -4.17 | -7.27 | -1.07 |
|  | Eraldo (2006) | -2.36 | | -4.95 | | 0.23 | **QRS duration (HBRP vs baseline)** | | | |  |
|  | Domenico (2012) | -1.10 | | -7.19 | | 4.99 |  | Eraldo, et al (2006) | -4.93 | -8.34 | -1.52 |
|  | Francesco (2008) | -2.52 | | -5.13 | | 0.09 |  | Kenneth, et al (2015) | 2.15 | -1.10 | 5.39 |
|  | Gianni (2014) | -2.68 | | -5.40 | | 0.04 |  | Domenico, et al (2012) | 4.27 | 1.05 | 7.50 |
|  | Mads (2014) | -2.37 | | -4.98 | | 0.24 |  | Gianni, et al (2014) | 18.42 | 15.02 | 21.82 |
|  | Combined | -2.42 | | -4.99 | | 0.16 |  | Mads, et al (2014) | 2.11 | -1.08 | 5.30 |
| **LVESV** | |  | |  | |  |  | Domenico, et al (2006) | 4.49 | 1.46 | 7.51 |
|  | Eraldo (2006) | 0.18 | | -2.78 | | 3.14 |  | Combined | 4.34 | 1.38 | 7.30 |
|  | Domenico (2012) | -2.18 | | -6.23 | | 1.88 | **QRS duration (HBRP vs RVAP)** | | | |  |
|  | Francesco (2008) | -0.31 | | -3.74 | | 3.12 |  | Eraldo, et al (2006) | -37.59 | -57.79 | -17.39 |
|  | Gianni (2014) | -0.34 | | -4.62 | | 3.93 |  | Kenneth, et al (2015) | -40.46 | -63.47 | -17.46 |
|  | Mads (2014) | 0.47 | | -2.54 | | 3.48 |  | Francesco, et al (2008) | -35.70 | -54.91 | -16.48 |
|  | Combined | -0.08 | | -2.98 | | 2.81 |  | Gianni, et al (2014) | -41.44 | -65.27 | -17.61 |
| **Inter-ventricular assynchrony** | | | | | | |  | Mads, et al (2014) | -40.86 | -63.58 | -18.15 |
|  | Eraldo (2006) | -17.46 | -28.00 | | -6.93 | |  | Domenico, et al (2012) | -49.23 | -57.97 | -40.49 |
|  | Domenico (2012) | -14.45 | -23.54 | | -5.36 | |  | Combined | -40.99 | -59.27 | -22.71 |
|  | Francesco (2008) | -19.11 | -29.86 | | -8.37 | | **Exposure time of fluoscopy** | | |  |  |
|  | Gianni (2014) | -19.03 | -30.23 | | -7.83 | |  | Kenneth, et al (2015) | 9.00 | 5.58 | 12.42 |
|  | Mads (2014) | -17.30 | -27.89 | | -6.72 | |  | Mads, et al (2014) | 2.70 | -0.61 | 6.01 |
|  | Domenico (2006) | -13.20 | -21.26 | | -5.14 | |  | Combined | 5.74 | 3.36 | 8.12 |
|  | Combined | -16.74 | -25.89 | | -7.59 | | **Lead impedence** | | |  |  |
| **NYHA class** | |  |  | |  | |  | Kenneth, et al (2015) | -79.49 | -107.36 | -51.62 |
|  | Eraldo (2006) | -0.14 | -0.42 | | 0.14 | |  | Domenico, et al (2012) | -106.98 | -141.48 | -72.47 |
|  | Francesco (2008) | -0.48 | -1.02 | | 0.05 | |  | Mads, et al (2014) | -84.67 | -112.57 | -56.77 |
|  | Mads (2014) | -0.41 | -1.14 | | 0.32 | |  | Combined | -88.21 | -112.42 | -64.00 |
|  | Combined | -0.35 | -0.81 | | 0.11 | | **Pth** | |  |  |  |
| **Mitral regurgitation** | | | | |  | |  | Eraldo, et al (2006) | 1.09 | 0.79 | 1.40 |
|  | Eraldo, et al (2006) | -0.96 | -1.98 | | 0.06 | |  | Kenneth, et al (2015) | 1.02 | 0.57 | 1.48 |
|  | Domenico, et al (2012) | -0.58 | -1.03 | | -0.13 | |  | Domenico, et al (2012) | 0.89 | 0.49 | 1.29 |
|  | Francesco, et al (2008) | -1.06 | -1.97 | | -0.16 | |  | Francesco, et al (2008) | 0.87 | 0.50 | 1.24 |
|  | Domenico, et al (2006) | -1.08 | -1.96 | | -0.20 | |  | Mads, et al (2014) | 0.91 | 0.52 | 1.30 |
|  | Combined | -0.93 | -1.65 | | -0.20 | |  | Combined | 0.96 | 0.62 | 1.30 |
| **MPI (Tei index)** | |  |  | |  | | **RWA** | |  |  |  |
|  | Domenico (2012) | -0.21 | -0.42 | | 0.01 | |  | Kenneth, et al (2015) | -5.20 | -7.49 | -2.91 |
|  | Gianni (2014) | -0.16 | -0.48 | | 0.15 | |  | Mads, et al (2014) | -6.90 | -8.55 | -5.25 |
|  | Domenico (2006) | -0.06 | -0.16 | | 0.03 | |  | Combined | -6.32 | -7.66 | -4.98 |
|  | Combined | -0.14 | -0.32 | | 0.04 | |  |  |  |  |  |

This table showed sensitivity analyses by the way of exclusion of 1 study at a time to determine the stability of the overall treatment effects (LVEF = left ventricular ejection fraction, LVEDV = left ventricular end diastolic volume, LVESV = left ventricular end systolic volume, NYHA = New York Heart Association, MPI = myocardial performance index, 6MWT = 6 minute walk test, PASP = pulmonary artery systolic pressure, Pth = pacing threshold, and RWA = R wave amplitude).
